# Human Cytomegalovirus (HCMV) Reactivation in the Mammary Gland Induces a Proinflammatory Cytokine Shift in Breast Milk

**DOI:** 10.3390/microorganisms8020289

**Published:** 2020-02-20

**Authors:** Tabea Rabe, Katrin Lazar, Christoffer Cambronero, Rangmar Goelz, Klaus Hamprecht

**Affiliations:** 1 Institute for Medical Virology and Epidemiology of Viral Diseases, University Hospital Tuebingen, 72076 Tuebingen, Germany; tabea.rabe@med.uni-tuebingen.de (T.R.); katrin.lazar@med.uni-tuebingen.de (K.L.); 2 Olink Proteomics, 75237 Uppsala, Sweden; christoffer.cambronero@olink.com; 3 Department of Neonatology, University Children’s Hospital Tuebingen, 72076 Tuebingen, Germany; rangmar.goelz@med.uni-tuebingen.de

**Keywords:** CMV, breastfeeding, neonates, chemokines, lactation

## Abstract

A locally restricted human cytomegalovirus (HCMV) reactivation in the mammary gland commonly occurs in nearly every IgG-seropositive breastfeeding mother. This unique phenomenon can therefore be used to study the reactivation process in an immunocompetent healthy host. Breast milk contains a variety of immunoactive compounds, including immune cells, antibodies, growth factors, and cytokines supporting the newborn’s immature immune system. To characterize the impact of HCMV reactivation on breast milk cytokines, we analyzed longitudinal breast milk samples of four IgG-seropositive and three IgG-seronegative mothers of preterm infants using Proximity Extension Assay (PEA) technology (Olink Proteomics, Uppsala, Sweden). Cytokine profiling revealed elevated cytokine levels in IgG-seropositive mothers’ milk whey. Reactivating mothers showed higher levels of CC-chemokines (MCP-2, CCL19, and CCL20) and CXC-chemokines (IL-8, CXCL9, CXCL10, and CXCL11), such as the proinflammatory cytokine IL-17C, glycoprotein CD5, and TNFSF14. HCMV reactivation seems to influence the cytokine profile in human breast milk. This work could open the door for further studies analyzing distinct relations of the cytokine network as well as phenotypical and functional T cell properties in background of HCMV DNA dynamics in early lactation.

## 1. Introduction

The interaction of a virus and its host’s immune system profoundly contributes to the phenomena of latency and reactivation of *Herpesviridae*. As HCMV reactivation in the mammary gland of lactating women is a highly frequent [1] and self-limited [2] process, analyzing the maternal immune response offers the possibility to observe locally restricted, virally induced immune reaction in a healthy immunocompetent host.

HCMV reactivation plays a major role in immunosuppressed recipients of hematopoietic stem cell or solid organ transplants via disseminated HCMV infection detected by viral DNAemia. In contrast, a locally restricted reactivation in the mammary gland occurs in nearly every healthy breastfeeding IgG-seropositive mother with HCMV shedding into milk in the absence of viral DNAemia [1]. Viral DNA load in milk whey (DNAlactia), which can be used to monitor viral shedding, mostly shows unimodal kinetics with an onset typically before day 10 postpartum [3]. Transmission via breast milk may lead to symptomatic HCMV infection in very preterm infants with a birth weight < 1500 g and a gestational age < 32 weeks [4].

Cytokines are polypeptides modulating innate and acquired immunity in networks by playing an important role in cell signaling and mediation of inflammatory responses [5]. In breast milk, cytokine levels typically show a high interindividual variability [6] and partly a decreasing tendency during the course of lactation [7,8]. Chemoattractants, anti-inflammatory cytokines, and other immunological components of breast milk may play a role in supporting the neonate’s immature immune system [9]. Whereas many factors influencing breast milk cytokines have been determined, [6,10,11,12] the impact of HCMV reactivation on cytokine profiles has not been demonstrated so far.

The exact mechanisms of viral reactivation in the mammary gland remain to be elucidated. In interstitial dendritic cells ex vivo, Interleukin-6 has been shown to be a potential key element for the reactivation of immediate early (IE) gene expression [13]. Additionally, other cytokines such as TNF-α seem to influence IE gene expression [14,15], which is necessary for initiation of the reactivation process.

In this study, we investigated differences in the longitudinal cytokine profile of HCMV IgG-seropositive and IgG-seronegative mothers’ milk whey to gain a first insight into the role of cytokines modulating the immune response to HCMV reactivation in the mammary gland.

## 2. Materials and Methods

### 2.1. Samples

Breast milk samples were obtained longitudinally from four IgG-seropositive and three IgG-seronegative breastfeeding mothers of preterm infants (Table 1) in four time ranges in weeks 2-3 (T1), 4-5 (T2), 6-7 (T3), and 8-9 (T4), postpartum. Five out of seven mothers (mothers 1-3, 6-7) were participants of the BlooMil study, whose defined time frames are published elsewhere [16]. For the quantification of CXCL10 concentrations in breast milk in an extended cohort, we reverted to the whole BlooMil study cohort, which included 18 seropositive and 10 seronegative breastfeeding mothers of mostly preterm infants at four time ranges after birth. The milk was processed as described earlier [16]. Fat- and cell-free milk whey, stored at -20°C for the measurement by proximity extension assay (PEA), and at -80°C for the measurement by enzyme-linked immunosorbent assay (ELISA), was used for analysis. Study samples were collected with written informed consent from all participating mothers, with approval of our institutional ethics committee (University Hospital Tuebingen: 804/2015BO2) and in accordance with the 1964 Helsinki declaration and its later amendments.

### 2.2. Proximity Extension Assay (PEA)

To investigate the levels of 92 inflammatory proteins, the Inflammation Panel of Olink Multiplex analysis service (Olink Proteomics, Uppsala, Sweden) was used.

The PEA method is based on the hybridization and extension of two complementary oligonucleotide sequences that are linked to different antibodies, each binding a specific region on the target protein. The results obtained by real-time PCR are given in normalized protein expression (NPX), a log2 scale unit calculated from normalized Ct (cycle threshold) values [17].

### 2.3. Enzyme-Linked Immunosorbent Assay (ELISA) for Cytokine Quantification

Human CXCL10/IP-10 DuoSet ELISA (R&D Systems, Minnesota, USA) was used to determine cytokine concentrations in pg/ml. Assays were performed in duplicates according to the manufacturer’s instructions. DuoSet ELISA (R&D Systems, Minnesota, USA) for CXCL10 was adjusted for the measurement of milk whey using 1.75 % nonfat dried milk powder (AppliChem GmbH, Darmstadt, Germany) in phosphate-buffered saline as reagent diluent, which made it possible to measure milk whey samples undiluted with a recovery rate of 99.3 % [range: 73.7 %; 120.0 %] (Table S1). Concentrations were obtained from a standard curve using a 4 Parameter Logistic Curve fit on GraphPad Prism (GraphPad Software inc., Version 8.1.0, San Diego, USA).

### 2.4. DNA Extraction and Determination of DNA Viral Load via qPCR

**Figure 1:**
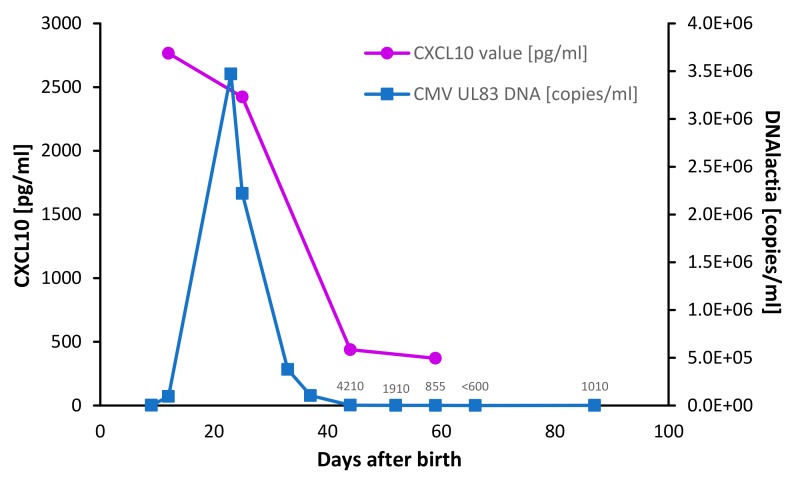
Exemplary course of viral shedding into breast milk during the first months of lactation (mother 3); cytomegalovirus (HCMV) DNA viral load, measured by qPCR targeting UL83 region, showed a unimodal course with a peak around day 23 (3 470 000 copies/ml); CXCL10 levels, measured by enzyme-linked immunosorbent assays (ELISA), showed a similar sharp decline like DNA viral load after viral load peak.

DNA was extracted using QIAcube DNA extractor with the QIAamp Blood Mini Kit from Qiagen (Hilden, Germany). DNAlactia was determined via quantitative real-time PCR targeting UL83 gene (CMV R-gene Kit, Biomérieux SA, Marcy-l’Etoile, France, lower limit of quantification: 600 copies/ml) using a LightCycler 2.0 (Roche, Basel, Switzerland). The DNA viral load of every participant of the BlooMil study was published by Lazar et al. [16]. Additional time points are included in Figure 1 and Table 1.

### 2.5. Statistics

Statistical analysis of data obtained by PEA was performed by Olink Statistical Service using linear mixed effect ANOVA (lmerTest package, R, Vienna, Austria) comparing IgG-seropositive and -negative mothers over time. P-values were adjusted for multiple testing (Benjamini-Hochberg). A post hoc test (lmerTest and emmeans package, R, Vienna, Austria) was performed for each significant assay result. Since proteins with less than 25 % detectability in our samples were excluded, 74 of 92 proteins remained for statistical analysis of the results obtained by PEA.

Longitudinal courses of CXCL10 measured by ELISA were statistically analyzed using a linear mixed model in SPSS (Version 25.0.0.1, IBM, Armonk, USA). Data on CXCL10 concentrations were logarithmically transformed to achieve normal distribution.

## 3. Results

Every IgG-seropositive mother whose milk whey was analyzed using PEA showed viral shedding into breast milk with a mean calculated onset on day 4.1 [range: 1.5; 5.5 days] after birth (Table 1).

An exemplary course of DNA viral load with a peak around day 23 (3.47*10^6^ copies/ml) and corresponding CXCL10 concentration is demonstrated (Figure 1). CXCL10 decreased in the course of lactation with a sharp decrease after viral load peak (Figure 1).

**Figure 2:**
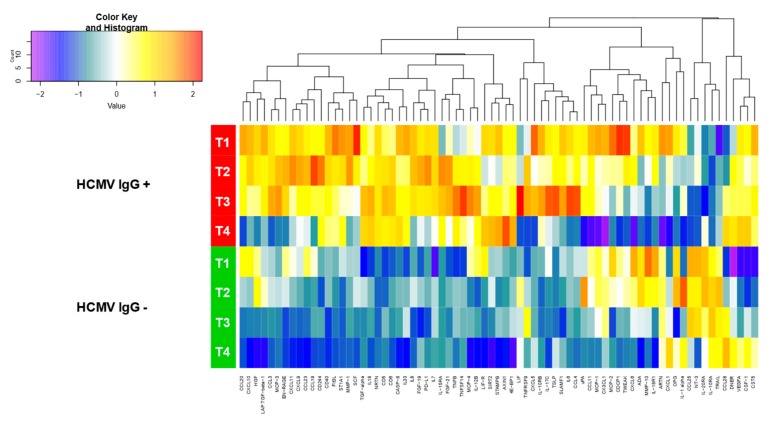
Heat map of longitudinal cytokine levels in milk whey of two index cases (mother 4, human cytomegalovirus (HCMV) IgG-seropositive, and mother 5, HCMV IgG-seronegative); obtained by Proximity Extension Assay technology; four time ranges at weeks 2-3 (T1), 4-5 (T2), 6-7 (T3), and 8-9 (T4), after birth.

**Figure A1:**
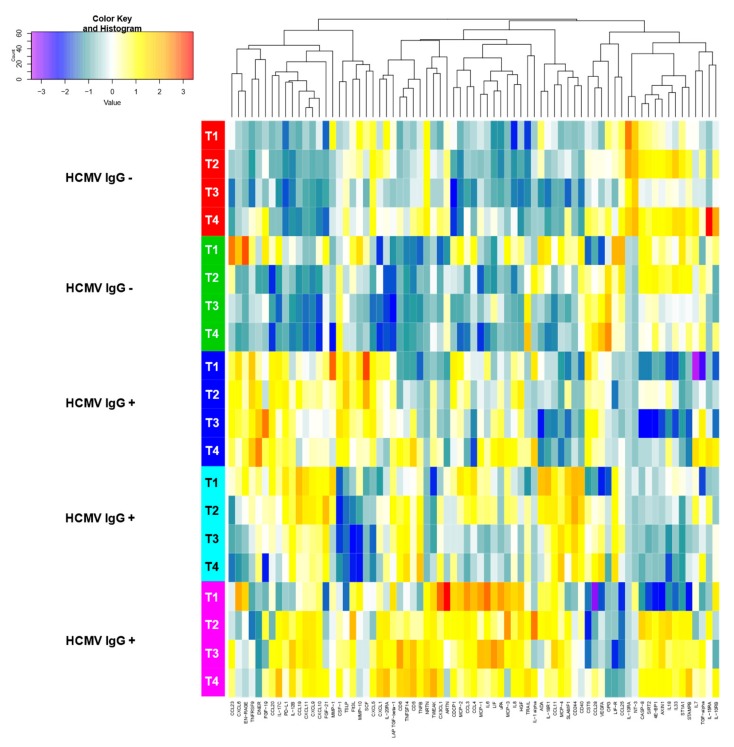
Heat map of longitudinal cytokine levels in milk whey of two IgG-seronegative and three IgG-seropositive mothers, obtained by Proximity Extension Assay technology. Four time ranges at weeks 2-3 (T1), 4-5 (T2), 6-7 (T3), and 8-9 (T4), after birth.

An exploratory visual analysis of our two index cases, the IgG-seropositive mother 4 and the IgG-seronegative mother 5, revealed a tendency towards increased cytokine levels in IgG-seropositive mothers’ milk whey as well as a general decreasing tendency particularly detectable from T3 to T4 for a multitude of the analyzed cytokines (Figure 2). This impression was underlined by further investigations involving two additional IgG-seronegative as well as three additional IgG-seropositive mothers. Furthermore, highly diverse cytokine levels were detectable in the seropositive cohort (Figure A1). Raw data are available in Table S2.

Statistically comparing IgG-seropositive and -negative samples regardless of the individual kinetics of each mother, milk whey of IgG-seropositive mothers showed significantly higher levels of CC-chemokines (MCP-2, CCL19, and CCL20) and CXC-chemokines (IL-8, CXCL9, CXCL10, and CXCL11) such as the proinflammatory cytokine IL-17C, glycoprotein CD5, and tumor necrosis factor superfamily member 14 (TNFSF14; Table 2). For all cytokines shown in Table 2, an overlap of the 95 % CI could be excluded.

**Figure 3:**
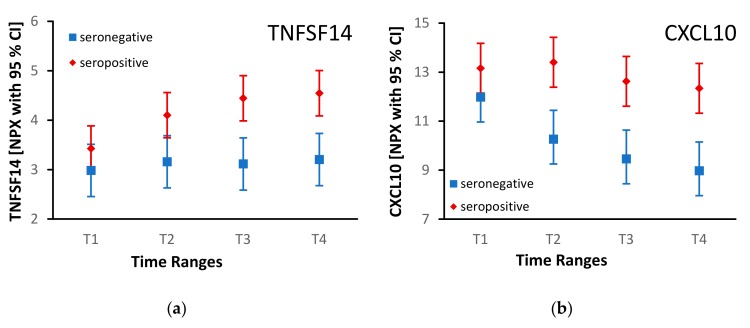
Longitudinal TNFSF14 (**a**) and CXCL10 (**b**) levels [Normalized Protein Expression; mean with 95 % confidence interval (CI)] in four IgG-seropositive (red) and three IgG-seronegative (blue) mothers‘ milk whey at four time ranges at weeks 2–3 (T1), 4–5 (T2), 6–7 (T3), and 8–9 (T4)

Of those proteins, CXCL10 and TNFSF14 levels also significantly changed differently over time depending on the serostatus (p=0.02 and p=0.02, respectively). TNFSF14 stayed on a constant level in IgG-seronegative and increased in IgG-seropositive mothers’ milk whey (Figure 3a). CXCL10 showed a generally decreasing tendency in both groups, but a later onset of decrease after the second time range (T2) regarding the IgG-seropositive group (Figure 3b). The peak viral load of mother 1-4 in milk whey was detected in the same time range (Table 1).

**Figure 4:**
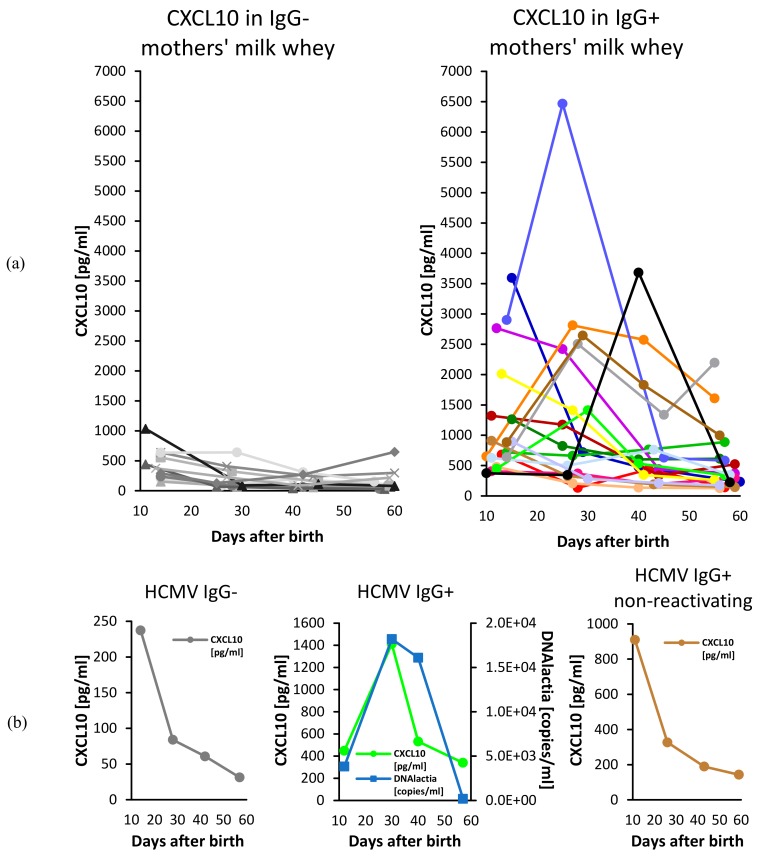
Longitudinal CXCL10 levels [pg/ml] in (**a**) 18 IgG-seropositive (right) and ten IgG-seronegative (left) mothers‘ milk wheys and (**b**) three individual courses of one seronegative, one seropositive, and one seropositive mother without HCMV shedding in breast milk. The three CXCL10 courses in (**b**) and Figure 1 can be attributed to individual courses in (**a**) by identical color coding.

Additional ELISAs for CXCL10 were performed in an extended cohort for the quantification of cytokine concentrations and the confirmation of the initial results (Figure 4a). Concentrations of CXCL10 significantly differed between seropositive and seronegative mothers’ milk whey (linear mixed model, p<0.001). The mean value [with 95% CI] of seropositive mothers’ milk whey (1004.3 pg/ml [747.0; 1261.5]) was four-fold higher than the mean value of seronegative mothers’ milk whey (248.1 pg/ml [176.2; 320.0]).

Three individual courses of CXCL10 in milk whey are presented in Figure 4b. Both the seronegative mother and the seropositive mother without observed HCMV reactivation showed a sharp decrease of CXCL10 in milk whey from the first (T1) to the second (T2) time range. A similar sharp decrease from T1 to T2 comprising more than half of the starting value could be seen in 6/10 (60 %) courses of the seronegative mothers, but only in 4/17 (23.5 %) of the seropositive and reactivating mothers (Figures S1–S3). Moreover, a (local) peak around peak viral load is observed in 6/17 (35.3 %) of the seropositive and reactivating mothers (Figure S3).

## 4. Discussion

This study is a first approach to investigate the effect of HCMV reactivation during lactation on breast milk cytokines and to gain an insight into the role of cytokines modulating the immune response to the viral reactivation process in breast milk. According to our results, a series of CC- and CXC-chemokines, including those induced by Interferon gamma (IFN-γ), such as CXCL10, might be released during the viral reactivation process in the mammary gland, which suggests an increased T_h_1 cell response. Chemokines play an important role in leukocyte migration to inflammation sites [18]. Their presence in breast milk and their increased expression during HCMV reactivation suggest the attraction and traffic of leukocytes into the mammary gland as part of the immune response. In our ongoing BlooMil study, a study on the role of T cell subsets during HCMV reactivation in breast milk, we investigate the cellular immune profile in HCMV reactivation during lactation in a well characterized study cohort. Breast milk cells in general were identified as potential sources of breast milk cytokines, as they express mRNA for MCP-1, IL-8, TGFß1, TGFß2, M-CSF, IL-6, and IL-1ß [6]. Since leukocytes only represent < 2 % of the total cell population in mature breast milk [19], the potential influence of nonimmune cells on breast milk cytokines should be considered as well. The predominant cell populations in breast milk are myoepithelial and luminal cells [20] of which the latter may contribute to CXCL10 production in breast milk [21]. Within breast milk leukocytes, there is evidence for an HCMV-pp65 CD8^+^ T cell response in reactivating mothers [22]. Additionally, it has been shown that CD14^+^ monocytes/macrophages may be an HCMV target cell population during viral reactivation [23]. There are many open questions about the origin of altered cytokine levels, specifically in seropositive mothers’ breast milk; further gene expression studies are required.

Special attention should be drawn to the increasing TNFSF14 levels in IgG-seropositive mothers’ milk whey (Figure 2). TNFSF14 is a ligand to the TNFRSF14, which is also used by Herpes simplex virus glycoprotein D as an entry mediator (HVEM) [24]. The binding of TNFSF14 and HVEM induces a costimulatory signal to T cells; in contrast, the binding of HVEM to the B- and T-lymphocyte attenuator (BTLA) induces an inhibitory signal to T cells [25,26]. HCMV expresses UL144, an orthologue to HVEM, which selectively binds BTLA facilitating inhibitory modulation of T cell proliferation as a part of the viral immune evasion strategy [25]. TNFSF14 signaling may be one path leading to enhanced T cell activity.

The CXC-chemokine CXCL10 typically decreases in the course of lactation [21]. We observed similar kinetics, especially in seronegative mothers’ milk whey (Figure 3b). Overall, in contrast to the most frequently unimodal HCMV DNA viral load, CXCL10 concentrations in seropositive mothers’ milk whey showed highly diverse patterns, partly decreasing throughout lactation, partly unimodal (Figure 4a and Figure S3). As shown in Figure 4b, the CXCL10 secretion pattern might be similar between seronegative mothers and non-reactivating seropositive mothers. However, the incidence of seropositive but not HCMV reactivating mothers is below 5 % [1], and therefore we are not able to confirm this phenomenon in our study cohort.

Increased plasma levels of CXCL10 were reported in context of HCMV infection of lung transplant recipients [27]. Various transcription factors are activated when a virus–cell contact occurs [28]. The nuclear factor kB (NF-kB) has been shown to be stimulated following HCMV infection; on the other hand, the NF-kB is involved in major immediate-early promoter (MIEP) transactivation [29]. More than 150 genes might be activated through this transcription factor, including those encoding cytokines and chemokines like IL-8, CXCL10, and CXCL11 [30]. Significantly enhanced levels of these cytokines might be the in vivo counterpart of protein analysis in the secretome of lytically HCMV infected endothelial cells and/or fibroblasts in vitro demonstrating secretion of CXCL9, CXCL10, CXCL11, MCP-2, and IL-8, among other cytokines [31].

A symptomatic HCMV infection of very preterm infants may lead to severe sepsis-like symptoms, including bradycardia, apnea, neutropenia, and thrombocytopenia [3,4]. For the prevention of postnatal HCMV infection, viral infectivity of breast milk from mothers with infants at risk (birth weight < 1500 g; gestational age < 32 weeks) is eliminated by short-term heat inactivation procedure based on the generation of a milk film in the neonatology department of the University Children’s Hospital Tuebingen [32,33,34]. Therefore, mother-to-infant HCMV transmissions in context of cytokine concentrations could not be evaluated. However, an interesting open question is the influence of short-term heat inactivation or common Holder pasteurization on cytokines and other immunological and nutritional important components of breast milk.

Measuring cytokine concentrations in breast milk is challenging, since breast milk is a dynamic body fluid that undergoes changes in composition throughout lactation but also shows high interindividual variation [35]. Therefore, we consider the small sample size as an undeniable limitation of the presented study. Due to the benefits of mother’s own milk for the neonate and the often lower availability of breast milk in mothers of preterm infants, longitudinal sample acquisition in a neonatal intensive care unit is particularly demanding [34]. However, the strengths can be seen in the longitudinal study course, which allows one to observe individual kinetics. As the composition of breast milk cytokines may differ between mothers of term and preterm infants [36], our study results may be limited by gestational age at delivery.

For the first time, this study demonstrated the potential impact of HCMV reactivation on breast milk cytokine profiles of mothers with preterm infants. Another open question is if cytokine release is not only a reaction to the virus, but if cytokines could even be involved already in the onset of viral shedding. The hypothesis that inflammation processes might be linked to viral reactivation can be found in various reports [14,37]. Indeed, especially colostrum contains a variety of immunomodulatory agents [8]. The concentration of IL-6, for example, was shown to be higher in breast milk compared to serum [10]. Keeping in mind that IL-6 supported MIEP activation [13], elevated cytokine concentration in breast milk compared to blood of defined cytokines might even contribute to HCMV reactivation, resulting in increased shedding of HCMV into milk.

## Figures and Tables

**Table 1:** Characteristics of the participating mothers.

Mother	Age	HCMV Serostatus	Gestational Age at Birth [Weeks + Days]	Onset of HCMV DNAlactia ^1^ [Day after Birth]	Peak Viral Load [Copies/ml] (Time Range ^2^)^3^
1	30	positive	30 + 2	5	183 000 (T2)
2	28	positive	33 + 2	5.5	11 000 (T2)
3	33	positive	24 + 2	4.5	3 470 000 (T2)
4	27	positive	30 + 6	1.5	238 000 (T2)
5	27	negative	26 + 3	-	-
6	31	negative	26 + 2	-	-
7	33	negative	24 + 5	-	-

^1^ estimated onset, determined using the arithmetic mean between last negative and first positive breast milk sample or birth date and first breast milk sample (if the first postnatal sample was already positive for viral DNA). ^2^ time range including peak viral load: week 2–3 (T1), week 4–5 (T2), week 6–7 (T3), and week 8–9 (T4). ^3^ Lazar et al. [16] and additional time points.

**Table 2:** Breast milk cytokine/chemokine concentrations [Normalized Protein Expression, Olink] —proteins with significant differences.

Category	Cytokines, Chemokines	Mean NPX Values with 95 % CI (IgG-Seropositive)	Mean NPX Values with 95 % CI (IgG-Seronegative)	P-Values ^1^
CC-chemokines	CCL19	7.6 [6.9; 8.3]	4.8 [4.0; 5.7]	6.28E-04
	CCL20	11.9 [11.5; 12.4]	10.7 [10.2; 11.2]	3.13E-03
	MCP-2	10.6 [10.2; 11.1]	9.5 [9.0; 10.0]	5.59E-03
CXC-chemokines	CXCL9	9.6 [8.6; 10.5]	5.9 [4.7; 7.0]	7.51E-04
	CXCL11	10.7 [9.8; 11.5]	7.6 [6.6; 8.6]	7.61E-04
	IL-8	9.6 [9.1; 10.2]	7.9 [7.3; 8.5]	1.47E-03
	CXCL10	12.9 [11.9; 13.8]	10.2 [9.1; 11.3]	3.19E-03
Others	IL-17C	4.5 [4.1; 4.8]	3.4 [3.0; 3.8]	2.37E-03
	CD5	1.9 [1.5; 2.3]	0.8 [0.3; 1.3]	3.97E-03
	TNFSF14	4.1 [3.7; 4.6]	3.1 [2.6; 3.6]	8.86E-03

^1^ Post hoc analysis for each significant assay result. P-values were calculated using the Tukey method.

## Supplementary Materials

The following are available online at https://www.mdpi.com/2076-2607/8/2/289/s1. Table S1: Spike/recovery testing for the measurement of CXCL10 in human milk whey; Table S2: Raw data on Proximity Extension Assay results; Figures S1–S3: Longitudinal CXCL10 courses in milk whey.
