# Supplementary material for: Human Cytomegalovirus (HCMV) Reactivation in the Mammary Gland Induces a Proinflammatory Cytokine Shift in Breast Milk
Source: Microorganisms. 2020 Feb 20;8(2):289. doi: 10.3390/microorganisms8020289 (PMC7074878; doi:10.3390/microorganisms8020289)
Supplement: Supplementary file 1 [file microorganisms-08-00289-s001.zip › microorganisms-722930-si-final/Figure S2.pdf]

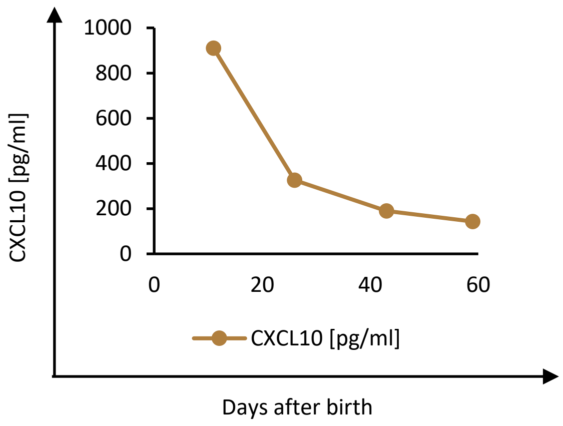

Figure S2: Longitudinal CXCL10 course in milk whey of a seropositive mother without HCMV reactivation during the observational period of two months postpartum. Color coding corresponds to Figure 4a.
