# Supplementary material for: Human Cytomegalovirus (HCMV) Reactivation in the Mammary Gland Induces a Proinflammatory Cytokine Shift in Breast Milk
Source: Microorganisms. 2020 Feb 20;8(2):289. doi: 10.3390/microorganisms8020289 (PMC7074878; doi:10.3390/microorganisms8020289)
Supplement: Supplementary file 1 [file microorganisms-08-00289-s001.zip › microorganisms-722930-si-final/Figure S3_Revisions.pdf]

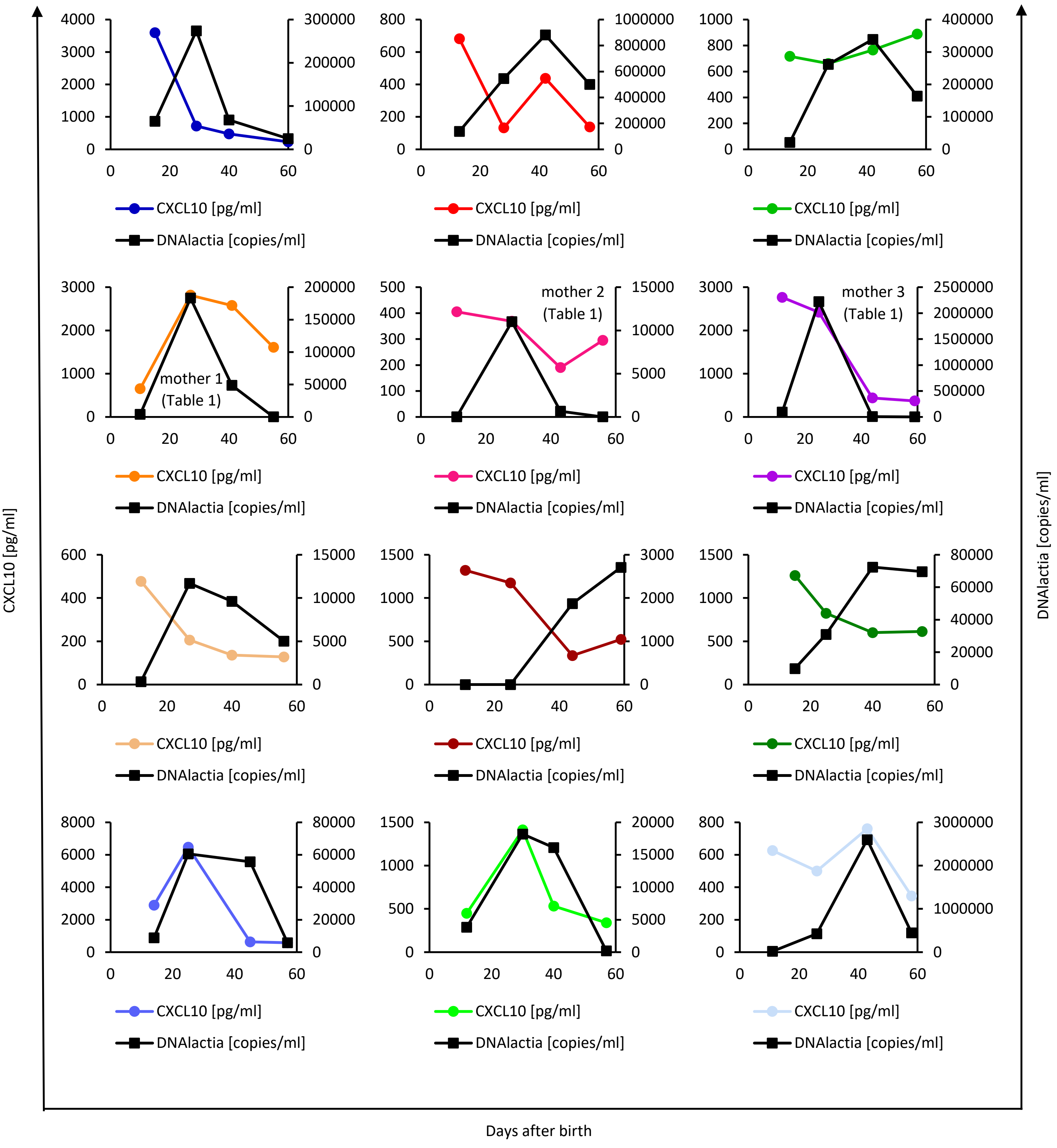
CXCL10 [pg/ml]
DNAlactia [copies/ml]
Days after birth
mother 1
(Table 1)
mother 2
(Table 1)
mother 3
(Table 1)
CXCL10 [pg/ml]
DNAlactia [copies/ml]

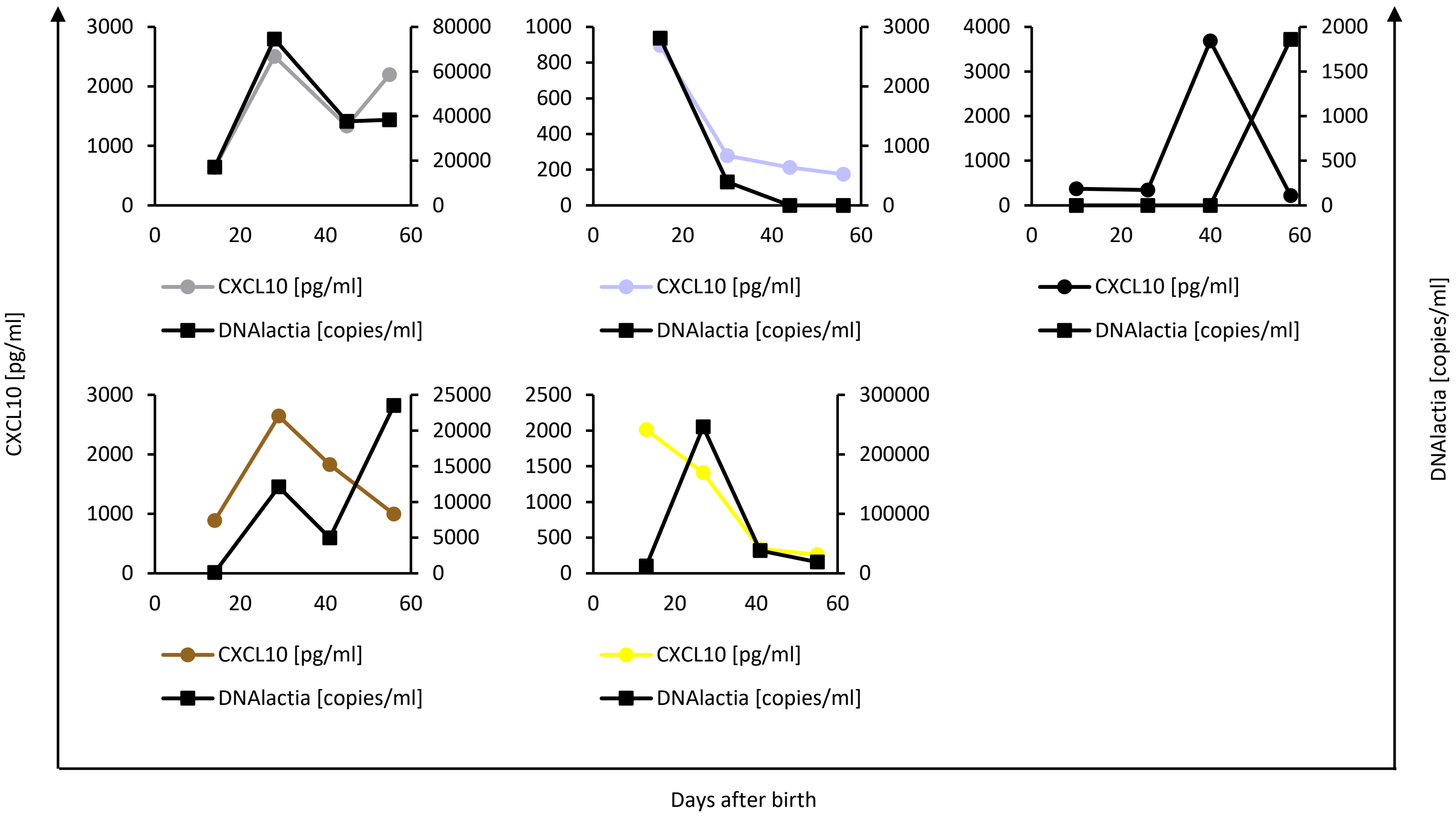

Figure S3: Longitudinal CXCL10 courses in context of HCMV DNA load [16] in milk whey of 17 seropositive and reactivating mothers during an observational period of two months postpartum. Color coding corresponds to Figure 4a.
